# Sinus Mucosa Thickness Changes and Ostium Involvement after Maxillary Sinus Floor Elevation in Sinus with Septa. A Cone Beam Computed Tomography Study

**DOI:** 10.3390/dj9080082

**Published:** 2021-07-21

**Authors:** Shingo Kato, Yuki Omori, Masatsugu Kanayama, Atsuya Hirota, Mauro Ferri, Karol Alí Apaza Alccayhuaman, Daniele Botticelli

**Affiliations:** 1 ARDEC Academy, 47923 Rimini, Italy; shingokato315@gmail.com (S.K.); ajtpgmgw.4649@gmail.com (M.K.); atsuyahirota@gmail.com (A.H.); daniele.botticelli@ardec.it (D.B.); 2 Department of Oral Implantology, Osaka Dental University, Osaka 573-1144, Japan; 3 ARDEC Foundation, Cartagena de Indias 130001, Colombia; medicina2000ctg@hotmail.com; 4 Department of Oral Biology, Medical University of Vienna, 1090 Vienna, Austria; caroline7_k@hotmail.com

**Keywords:** maxillary sinus, cone-beam computed tomography, sinus mucosa, Schneiderian membrane, maxillary sinus ostium

## Abstract

Background: A thickening of the sinus mucosa is observed after sinus floor augmentation. The objective of this retrospective study was to evaluate the influence of the presence of septa in the dimensional variation and ostium involvement over time of the Schneiderian mucosa after sinus floor augmentation. Materials and Methods: Fifteen sinuses with septa (septa group) and 15 without (control group) were selected. CBCTs taken before surgery, and were analyzed after 1 week and after 9 months. Schneiderian membrane thickness changes over time and involvement of the ostium were evaluated. Results: Four perforations occurred in the septa group and none in the control group. After 1 week of healing, the sinus mucosa thickness increased in height by 5.7 mm and 7.1 mm in the septa and control groups, respectively. In this period, the patency of the ostium decreased in both groups, and three infundibula were obstructed in the septa group, and five in the control group. The mucosa was thicker and the edema was closer to the ostium in the control compared to in the septa group. After 9 months of healing, the dimensions regressed to normal pattern and no obstruction of the infundibula were observed. No statistically significant differences were found between septa and control groups. Conclusions: after one week of healing, the sinus mucosa increased in dimensions in both septa and control groups. However, the sinus mucosa presented a tendency of being thicker and closer to the ostium, resulting in a higher number of infundibula obstructions, in the control group compared to in the septa group. After 9 months, the sinus mucosa regressed to normal dimensions and no obstructions of the infundibula were observed in any group.

## 1. Introduction

Maxillary sinus floor elevation is nowadays considered a safe and effective surgical technique to allow the prosthetic restauration supported by implants in an atrophic posterior region of the maxilla [1]. Nevertheless, various anatomical conditions must be considered when applying this approach [2]. Excluding the presence of sinus pathology that might contra-indicate sinus surgery, the height of the residual bone crest defines the limits to which this technique is indicated, while the width of the lateral wall of the sinus might make the preparation of the antrostomy difficult [2]. The distance between the palatal-nasal recess (PNR) and the floor of the sinus, as well as the angle formed between the palatal and nasal walls in this region, may also influence the prognosis. In fact, an angle of the PNR <90° is considered a risk factor for perforation during mucosa elevation [3]. The position of the posterior superior alveolar artery may influence the position of the antrostomy [4,5]. Moreover, in case of lesion of the artery, hemorrhagic events may seriously complicate the surgery [6].

The presence of septa might also influence the surgical approach and generate perforations of the mucosa during the elevation procedures [7,8,9,10].

Other aspects that should be evaluated are the dimensions of the ostium and patency of the infundibulum [2,11]. This aspect is truly relevant because the edema and bleeding after the surgery will increase the dimensions of the sinus mucosa, as documented in experimental [12,13], pilot [14,15], retrospective [16] and prospective studies [17,18], as well as in randomized controlled trials [4,5,19,20]. However, the association between the presence of septa and the changes in dimensions of the sinus mucosa after sinus lifting and the involvement of the ostium and infundibulum has not yet been documented. Hence, the aim of the present study was to evaluate the influence of the presence of septa in the dimensional variation and ostium involvement over time of the Schneiderian mucosa after sinus floor augmentation.

## 2. Materials and Methods

The records of patients included in randomized controlled trials (RCT) [4,5,19,20] performed at the University Corporation Rafael Núñez, Cartagena de Indias, Colombia were further evaluated after the approval for supplementary assessments of the data by the local ethical committee (protocol # CURN-0002-CE282020).

The following inclusion criteria had to be fulfilled by the patients to be included in the RCTs mentioned above: (i) presence of an edentulous region in the posterior region of the maxilla; (ii) height of the bone crest ≤4 mm; (iii) ≥21 years of age; (iv) good general health; (v) not pregnant. An additional analysis of the same set of data was performed with the following inclusion criteria: (i) treated for sinus floor elevation with a lateral access; (ii) presence of septa that might interfere with the surgery within the maxillary sinus; (iii) availability of good quality cone beam computed tomographies (CBCTs) before surgery (T0), and after 1 week (T1w) and 9 months (T9m). As control group, a similar number of sinuses from different patients treated for sinus floor elevation with a lateral access were randomly selected among the group without septa.

**Figure 1:**
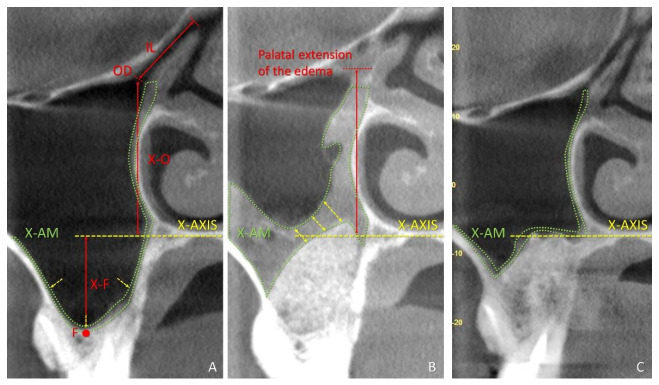
Coronal view of tomographic images of a maxillary sinus before surgery (**A**), and after 1 week (**B**) and 9 months (**C**). X-axis, nose floor; X-AM, area of the sinus mucosa (delimited by dotted green lines); IL, infundibulum length; OD, maxillary sinus ostium diameter; F, sinus floor; X-F, distance between X-axis and F; X-O, distance between X-axis and maxillary sinus ostium. Yellow arrows, positions of the measurements of the thickness of the mucosa. Note the loss of patency at ostium and infundibulum after 1 week from sinus floor elevation.

As references for the coronal view, the x-axis, drawn at the base of the nose, and the base of the sinus floor were used (Figure 1). The following assessments were performed in the coronal view of the CBCTs taken at T0: (i) the distance between the maxillary sinus ostium (MSO) and the sinus floor; (ii) the MSO diameter; (iii) the infundibulum length; (iv) the number of infundibula out of patency; (v) the sinus mucosa thickness [21]. 

The following assessments were performed in the coronal view of the CBCTs taken at T1w and T9m: (i) the MSO diameter; (ii) the number of infundibula out of patency; (iii) the sinus mucosa thickness; (iv) the distance between ostium and edema.

In the lateral view, the vertical extension of the edema above (+) or below (−) an axis drawn at the base of the nose (Z-axis) was measured at T1w and T9m.

The height of the septa and the distance between the ostium and the septum were also measured. Moreover, the Irinakis classification of the septa was adopted [8]. This classification basically includes the following categories according to the orientation of the septa: Class I, buccal-lingual direction; Class II, mesial-distal direction; Class III, horizontal orientation; Class IV: septum of a combination of Class I, II, or III.

All CBCTs were taken using a 3D Accuitomo 170 Tomograph (J Morita Corporation, Kyoto, Japan). The CBCT measurements were performed with the i-Dixel 2.0 software (J. Morita Corporation, Kyoto, Japan).

### Data Analyses

The main variables were sinus height and ostium diameter. A Mann–Whitney test was applied to evaluate the differences between the septa group and the control group. As an explorative aim, the differences between periods were also evaluated for all parameters using a Wilcoxon test. The analyses were performed with the IBM SPSS Statistics software v19.0 (IBM Inc., Chicago, IL, USA).

## 3. Results

### 3.1. Clinical Report

The CBCTs of eighty-eight sinuses of seventy-five patients were assessed. Fifteen sinuses from 12 patients satisfied the inclusion criteria (mean age 55.2 ± 9.4 years). Fifteen randomly selected patients (mean age 57.8 ± 8.1 years) without septa in the maxillary sinus were included in the control group (Table 1). Ten out of thirty sinuses were located at the right side, while twenty were at the left side.

At the analysis of the records, a lateral access window was adopted for all patients included in the study. It was observed that 10 sinuses in each group received OsteoBiol Gen-Os^®^, 250–1000 µm (Tecnoss, Giaveno, Italy) as filler material and a collagen membrane OsteoBiol^®^ Evolution, 0.3 mm (Tecnoss, Giaveno, Italy) to protect the antrostomy. Five sinuses in each group received Cerabone granulate 1.0–2.0 mm (Botiss Biomaterials GmbH, Zossen, Germany) as filler material, while the antrostomy was protected with Collprotect^®^ collagen membrane (Botiss Biomaterials GmbH).

Four perforations were reported during surgery in the septa group (26.7%), all protected with a collagen membrane, while no perforations were reported in the control group. The perforations were about 1, 2, 3 and 3 × 5 mm in dimensions, respectively. No unexpected complications were reported.

### 3.2. CBCTs Assessments

**Figure 2:**
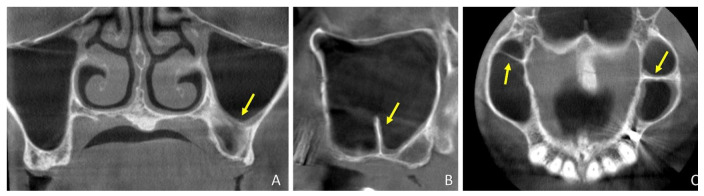
CBCT images illustrating the situation before surgery of a septa sinus. The yellow arrows indicate a septum in the left sinus in the (**A**) coronal, (**B**) lateral, and (**C**) axial views. In C, a septum is visible also in the right sinus.

At the baseline, the septa presented a mean height of 6.3 ± 2.4 mm (min 2.9 mm; max 9.8 mm) and were mainly positioned in the molar region (Figure 2A,B,C), 9.6 ± 4.9 mm distally to the ostium. According to the Irinakis classification [8], eleven septa were Class I, one Class II, and three Class IV. Ten sinuses presented one single septum, three presented two septa, and two presented three septa.

The ostia were mainly located in the premolar region, but some ostia could be found in the molar region. In the control group, they were located more distally compared to the septa group (Table 1). The distance between the ostium (MSO) and the base of the sinus was 33.2 ± 3.9 mm in the septa group and 32.6 ± 4.6 mm in the control group. The MSO diameter was 1.7 ± 0.4 mm and 2.1 ± 0.8 mm in the septa and the control group (Table 2). The respective infundibulum lengths were 8.5 ± 1.3 mm and 9.2 ± 2.1 mm. No obstructions of the infundibula were found before surgery. The mucosa thickness was 1.1 ± 0.7 mm and 1.0 ± 0.7 mm, respectively. None of the differences between the septa and control groups was statistically significant.

**Figure 3:**
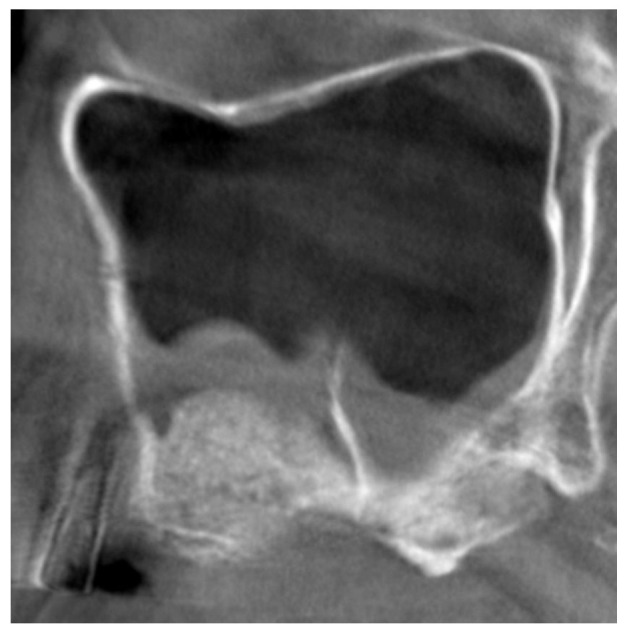
Lateral view of the case in Figure 1, 1 week after the sinus floor elevation. The sinus mucosa showed an increase in dimensions above anteriorly and posteriorly the septum.

After 1 week of healing (Table 2), the MSO diameter decreased in both groups to 1.0 ± 0.6 mm in the septa group, and to 1.0 ± 0.8 mm in the control group, while the sinus mucosa width increased to 6.7 ± 7.8 mm and to 8.2 ± 7.0 mm in the septa and control group, respectively (Figure 3). 

The distance between the extension of the edema to the ostium was 11.8 ± 10.7 mm in the septa group, and 7.5 ± 8.8 mm in the control group (*p* = 0.310). Three obstructions of the infundibula in the septa group (20%) and five in the control group (33.3%) were found. None of the differences between the septa and control groups was statistically significant.

Nine months after the surgery (Table 2), the MSO diameter returned to about the same diameter in both groups, as well as the mucosa width in the septa group. However, in the control group, the mucosa presented a higher width compared to the dimensions before surgery. None of the differences between the septa and control groups was statistically significant excluding the difference in mucosa thickness (*p* = 0.028) and mucosa area (*p* = 0.029) in the coronal view. The distance of the edema from the ostium decreased both in the coronal and in the lateral view (Table 3) in both groups. No obstructions of any infundibula were found.

## 4. Discussion

The aim of the present study was to evaluate the influence of the presence of septa in the dimensional variation over time of the Schneiderian mucosa after sinus floor augmentation. The sinuses of the control group presented a tendency of a higher increase in the mucosa width and involvement of the ostium.

The mean height of the septa in the present study was 5.7 mm, dimension within the range of the height reported in a review on maxillary sinus septa [22]. The location of the septa was mainly at the level of the first and second molars, with the data being in agreement with those described in a clinical study [23].

The initial width of the sinus mucosa was 1.1 mm in the septa group and 1.0 in the control group; dimension slight smaller than those reported in another study in which the evaluation was performed in patients also scheduled for sinus floor elevation [24]. In that study, the width of the sinus mucosa was 2 mm. It has to be considered, however, that the width of the sinus mucosa may vary depending on the region where the assessment is performed [25]. Moreover, the presence of periapical lesions, incongruous endodontic treatment, severe caries and periodontal bone loss increases the width of the sinus mucosa [26]. The extraction of teeth presenting such pathologies might reduce, but not completely resolve, the thickening of the sinus mucosa [26,27].

In the sinuses of both groups, one week after the surgery, a transient increase in the thickness of the sinus mucosa was observed. This is due to the clot/edema that is formed after the surgery so that it should be considered a virtual increase in the sinus mucosa thickness [21]. Various studies described the increasing in the thickness of the sinus mucosa after sinus floor elevation. In an experimental study in monkeys [12], edema and clot were seen both macroscopically and histologically during the first periods of healing. The swelling regressed completely within a month. It was shown that the thickness of the sinus mucosa increases progressively during the first week of haling after sinus floor elevation applying a lateral access [18]. The return to normality of the dimensions of the sinus mucosa has been reported to occur within 3 weeks in the transcrestal approach [14] or few months in the lateral access [16,17,18,19,20]. It has been also shown that the increase in mucosa thickness after sinus floor elevation is directly correlated to the dimensions of the antrostomies [5]. In the present study, a higher increase in the mucosa thickness was seen in the control compared to the septa group. Even though the difference was not statistically significant, a difference of 1.5 mm in thickness might mean that the presence of the septa may limit the extension of the edema, as confirmed by other data reported in the present study. In fact, the extension of the edema on the palatal wall one week after the surgery was 4.3 mm closer to the ostium in the control group compared to the septa group. Indeed, the edema also involved the ostium as shown by the higher number of obstructions of the infundibula in the control group (5 events) compared to the septa group (3 events). Nevertheless, none of these cases reported clinical complications. This agrees with a clinical study [17], in which the initial sinus mucosa thickness was 1.9 mm. Seven infundibula in 53 CBCTs were out of patency before sinus floor elevation. After surgery, the mucosa increased to about 4 mm, and 16 infundibula were obstructed. The width returned to normality after 7.5 months. However, five obstruction were still observed.

In the present study, after 9 months of healing, the sinus mucosa returned to normality in the septa group. In the control group, however, the sinus mucosa was 1.2 mm thicker compared to T0. This was mainly due to a single case that still presented a thick sinus mucosa. This sinus, however, did not show any pathological appearance, nor did the patient refer any symptom. At this stage of healing, no obstructions of any infundibula were observed, and all sinuses were devoid of pathologies.

Four perforations (26.7%) were reported in the septa group; an event that did not occur in the control group. Nevertheless, the dimensions of the perforations were small so that the placement of a collagen membrane subjacent the sinus mucosa allowed all surgeries to be successfully accomplished. The presence of septa exposes to a higher risk of sinus mucosa perforations, as reported in various studies [7,8,9,10]. In a retrospective clinical study [8], the outcomes of 79 consecutive sinus floor elevations with lateral window approach were evaluated. Interfering septa were found in about 48% of sinuses. Seventeen perforations (44.7%) were recorded at the sinuses with septa, while only one perforation occurred at the sinus without septa.

As limitations of the present study, the 2-dimensional analysis performed and the retrospective design should be included. Another limitation is represented by the difficulties to discriminate on CBCT between the real sinus mucosa thickness and thickening generated by the edema. For this reason, the term virtual sinus mucosa thickening was introduced [21]. A larger sample might allow statistically significant differences between groups to be found, especially in relation to sinus mucosa thickening and extension of the edema toward the ostium.

## 5. Conclusions

In conclusion, after one week of healing, the sinus mucosa increased in dimensions in both septa and control groups. However, the sinus mucosa presented a tendency of being thicker and closer to the ostium, resulting in a higher number of infundibula obstructions in the control group compared to in the septa group. After 9 months, the sinus mucosa regressed to normal dimensions and no obstructions of the infundibula were observed in any group.

## Figures and Tables

**Table 1:** Demographic and clinical data. Sinuses n = 15.

	Gender	Age (Years)	Smokers	Sinus Side	Type of Edentulism	Ostium Position	Septa Position
SEPTA	9 females 3 males	55.2 ± 9.4	NONE	7 right; 8 left	10 partial, 5 total	9 PM1, 4 PM2, 2 M1	1 PM2, 3 M1, 11 M2
CONTROL	8 females 7 males	57.8 ± 8.1	NONE	3 right; 12 left	13 partial, 2 total	2 PM1, 7 PM2, 6 M1	
PM1, PM2 = First or second premolar regions; M1, M2 = First or second molar regions.							

**Table 2:** Radiographic anatomical measures in the coronal view of CBCTs taken at T0 (before surgery), at T1w (1 week after surgery), and at T9m (9 months after surgery). Data in millimeters or square millimeters only for the mucosa area.

		MSO to X-Axis	MSO to Sinus Floor	Infundibulum Length	MSO Diameter	Number of Obstructions	Mucosa Thickness	Mucosa Area	Distance between Ostium and Edema
SEPTA Mean values ± SD Minimum; Maximum	T0	24.2 ± 3.4 18.5; 31.1	33.2 ± 3.9 25.2; 38.4	8.5 ± 1.3 6.3; 10.8	1.7 ± 0.4 ^b^ 1.3; 2.7	0	1.1 ± 0.7 ^b^ 0.3; 2.3	13.2 ± 8.3 ^b^ 5.6; 27.1	-
	T1w		-	-	1.0 ± 0.6 ^b,c^ 0.0; 2.2	3 (20%)	6.7 ± 7.8 ^b,c^ 0.5; 31.2	168.7 ± 161.0 ^b.c^ 7.9; 576.4	11.8 ± 10.7 ^c^ −1.4; 29.5
	T9m		-	-	1.6 ± 0.4 ^c^ 1.3; 2.2	0	0.9 ± 0.6 ^a,c^ 0.5; 2.4	14.3 ± 7.3 ^a,c^ 10.0; 27.8	24.6 ± 6.0 ^c^ 9.8; 31.9
CONTROL Mean values ± SD Minimum; Maximum	T0	24.9 ± 4.1 13.7; 30.0	32.6 ± 4.6 26.3; 41	9.2 ± 2.1 7.0; 13.6	2.1 ± 0.8 ^b^ 1.2; 4.6	0	1.0 ± 0.7 ^b^ 0.5; 2.4	14.7 ± 15.7 ^b^ 5.4; 66.3	-
	T1w		-	-	1.0 ± 0.8 ^b,c^ 0.0; 2.5	5 (33.3%)	8.2 ± 7.0 ^b,c^ 0.9; 24.1	199.9 ± 135.7 ^b,c^ 11.3; 449.5	7.5 ± 8.8 ^c^ 1.5; 24.7
	T9m		-	-	1.6 ± 0.7 ^c^ 0.6; 2.9	0	2.3 ± 2.8 ^a,c^ 0.6; 11.4	44.0 ± 69.1 ^a,c^ 8.7; 283.0	21.5 ± 8.0 ^c^ 11.7; 34.7

SD, Standard deviation. MSO, Maxillary sinus ostium. ^a^ = *p* < 0.05 between septa and control groups; ^b^ = *p* < 0.05 between T0 and T1w; ^c^ = *p* < 0.05 between T1 and T9w. No differences between T0 and T9m.

**Table 3:** Radiographic anatomical measures in the lateral view of CBCTs taken at T0 (before surgery), at T1w (1 week after surgery), and at T9m (9 months after surgery). Data in millimeters or square millimeters only for the virtual mucosa area.

		Mucosa Area	Mesial Vertical Extension above (+) or below (−) the Z-Axis	Distal Vertical Extension above (+) or below (−) the Z-Axis
SEPTA Mean values ± SD Minimum; Maximum	T0	29.8 ± 30.7 ^b^ 15.8; 122.9	-	-
	T1w	297.7 ± 262.0 ^b,c^ 11.8; 875.7	10.8 ± 9.1 ^c^ −2.8; 25.9	14.2 ± 10.4 2.6; 30.4
	T9m	41.2 ± 41.6 ^c^ 9.7; 157.9	1.7 ± 4.0 ^c^ −2.7; 8.3	0.7 ± 4.0 −2.8; 12.4
CONTROL Mean values ± SD Minimum; Maximum	T0	26.7 ± 20.0 ^b^ 11.8; 83.3	-	-
	T1w	311.5 ± 205.3 ^b,c^ 21.5; 585.9	14.1 ± 10.8 ^c^ −5.7; 30.0	12.8 ± 11.1 −7.0; 36.0
	T9m	82.4 ± 144.8 ^c^ 14.2; 575.9	2.8 ± 6.9 ^c^ −5.9; 18.4	2.2 ± 6.1 −7.5; 19.0

SD, Standard deviation. ^b^ = *p* < 0.05 between T0 and T1w; ^c^ = *p* < 0.05 between T1 and T9w. No statistically significant differences were found between the septa and control groups and between T0 and T9m in both groups.

## Data Availability

The data are available on reasonable request.
